# Mechanical circulatory support in ventricular arrhythmias

**DOI:** 10.3389/fcvm.2022.987008

**Published:** 2022-10-11

**Authors:** Guido Tavazzi, Valentino Dammassa, Costanza Natalia Julia Colombo, Eloisa Arbustini, Thomas Castelein, Martin Balik, Christophe Vandenbriele

**Affiliations:** ^1^Department of Clinical, Surgical, Diagnostic and Paediatric Sciences, University of Pavia, Pavia, Italy; ^2^Department of Anaesthesia, Intensive Care and Pain Therapy, Fondazione IRCCS Policlinico San Matteo, Pavia, Italy; ^3^PhD in Experimental Medicine, University of Pavia, Pavia, Italy; ^4^Adult Intensive Care Unit, Royal Brompton Hospital, London, United Kingdom; ^5^Centre for Inherited Cardiovascular Diseases, Fondazione IRCCS Policlinico San Matteo, Pavia, Italy; ^6^Cardiovascular Center, Onze-Lieve-Vrouwziekenhuis Hospital, Aalst, Belgium; ^7^Department of Anesthesiology and Intensive Care, First Medical Faculty and General University Hospital, Charles University in Prague, Prague, Czechia; ^8^Department of Cardiovascular Diseases, University Hospitals Leuven, Leuven, Belgium; ^9^Department of Cardiovascular Sciences, KU Leuven, Leuven, Belgium

**Keywords:** mechanical circulatory support (MCS), arrhythmias, extracorporeal membrane oxygenation (ECMO), hemodynamic, review

## Abstract

In atrial and ventricular tachyarrhythmias, reduced time for ventricular filling and loss of atrial contribution lead to a significant reduction in cardiac output, resulting in cardiogenic shock. This may also occur during catheter ablation in 11% of overall procedures and is associated with increased mortality. Managing cardiogenic shock and (supra) ventricular arrhythmias is particularly challenging. Inotropic support may exacerbate tachyarrhythmias or accelerate heart rate; antiarrhythmic drugs often come with negative inotropic effects, and electrical reconversions may risk worsening circulatory failure or even cardiac arrest. The drop in native cardiac output during an arrhythmic storm can be partly covered by the insertion of percutaneous mechanical circulatory support (MCS) devices guaranteeing end-organ perfusion. This provides physicians a time window of stability to investigate the underlying cause of arrhythmia and allow proper therapeutic interventions (e.g., percutaneous coronary intervention and catheter ablation). Temporary MCS can be used in the case of overt hemodynamic decompensation or as a “preemptive strategy” to avoid circulatory instability during interventional cardiology procedures in high-risk patients. Despite the increasing use of MCS in cardiogenic shock and during catheter ablation procedures, the recommendation level is still low, considering the lack of large observational studies and randomized clinical trials. Therefore, the evidence on the timing and the kinds of MCS devices has also scarcely been investigated. In the current review, we discuss the available evidence in the literature and gaps in knowledge on the use of MCS devices in the setting of ventricular arrhythmias and arrhythmic storms, including a specific focus on pathophysiology and related therapies.

## Introduction

Ventricular arrhythmias are responsible for a significant number of sudden cardiac deaths (SCD) (1). Implantable cardioverter defibrillators (ICD) have been extensively proven to be superior to antiarrhythmic drugs in preventing ventricular arrhythmias in high-risk patients (2–4). Nonetheless, ICDs do not prevent the recurrence of ventricular arrhythmias; even when necessary and lifesaving, shocks have a severe impact on quality of life (5).

Heart failure with reduced ejection fraction is burdened with a higher risk of SCD and poor prognosis. Left ventricular ejection fraction (LVEF) has been recognized as the strongest predictor of ventricular arrhythmias and mortality in patients with cardiomyopathies (6), and it represents the main criterion in the decision-making process when considering the implantation of an ICD as primary prevention (7). Reduced LVEF was also found to be an independent predictor of ventricular arrhythmia recurrence in patients with ischaemic heart disease and ICD as secondary prevention (8). Nonetheless, the prediction of SCD still represents a clinical challenge for cardiologists. In the future, the selection of candidates may not only rely on echocardiography-derived LVEF but on multiparametric imaging, including cardiac magnetic resonance and strain echocardiography (9).

Catheter ablation (CA) of ventricular tachycardia (VT) represents a percutaneous technique that can permanently treat VT and prevent its recurrence. Current expert consensus recommends using CA for recurrent VT refractory to antiarrhythmic therapy or in those who tolerate antiarrhythmic drugs poorly (10). Preprocedural planning, mainly based on 12-lead ECG findings, is a fundamental step given the choice of mapping and ablation strategies. To achieve a successful ablation, four different strategies have been developed to map VT: activation mapping, entrainment mapping, pace mapping, and substrate mapping. Each of these techniques has its own advantages and applications in a specific context based on the arrhythmogenic mechanism and the hemodynamic tolerance of ventricular arrhythmia (11).

Patients requiring CA for ventricular arrhythmias may present with structural heart disease, commonly ischemic heart disease. In such a clinical scenario, acute hemodynamic decompensation during the CA procedure is not uncommon, affecting 11% of patients and is associated with an increased mortality rate (12). In addition, the coexistence of structural heart disease reduces the hemodynamic tolerance to the onset of VT episodes, making activation and entrainment mapping unfeasible and unsafe in patients without cardiovascular support. The use of general anesthesia, particularly if prolonged, for CA procedures further increases the risk of cardiovascular decompensation. Other clinical factors associated with acute hemodynamic decompensation during CA are advanced age, the presence of comorbidities (diabetes mellitus and chronic obstructive pulmonary disease), and presentation with VT storm (12).

Mechanical circulatory support (MCS) devices, such as Impella and venoarterial extracorporeal membrane oxygenation (VA ECMO), may represent an appealing tool to support blood pressure and guarantee adequate end-organ perfusion during the CA of VT. However, MCS implantation carries its own costs and intrinsic risk of complications, mainly related to bleeding and vascular complications (13, 14). For these reasons, the selection of ideal candidates for MCS insertion during CA is fundamental, especially if a preemptive insertion of these devices is considered to increase the safety of the CA procedure (15, 16).

Despite the increasing use of MCS during CA of ventricular arrhythmias, both as rescue therapy for the onset of acute hemodynamic decompensation and as a prophylactic strategy to avoid cardiovascular instability, robust evidence deriving from large randomized clinical trials is missing, and most of the current knowledge is based on the experience of specialized centers. A recently published systematic review by Mariani *et al*. analyzed the available evidence regarding the use of temporary MCS in life-threatening arrhythmias with interesting conclusions about the application of the PAINESD risk score, as further discussed, and the prophylactic use of VA ECMO for an electrical storms (17). Nonetheless, a significant lack of knowledge still exists regarding patient and device selection and ideal timing for implantation during CA for VT and electrical storms.

This review aims to provide an overview of the existing literature on MCS in patients with ventricular arrhythmias and arrhythmic storms, highlighting the benefits and gaps in knowledge for each therapeutic strategy.

## Percutaneous MCS support during arrhythmia-related cardiogenic shock

Cardiac output (CO) is determined by the heart rate (HR) and by the stroke volume (SV) of the left ventricle (LV). The latter is the difference between end-diastolic (EDV) and end-systolic volume (ESV).

- CO = HR ^*^ SV
- SV = EDV – ESV

**Figure 1:**
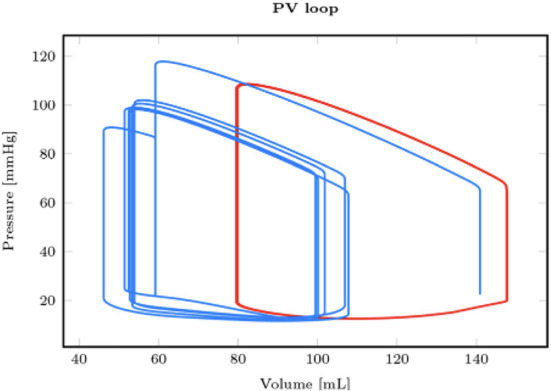
Comparison between a reference healthy pressure-volume loop in sinus rhythm (red) and the one obtained in hemodynamically tolerated ventricular tachycardia [reproduced with permission from (19)].

From the first equation, it is easy to understand that (extreme) bradycardia will lead to decreased cardiac output and eventual low-output cardiogenic shock (CS) (18). In parallel, atrial, and ventricular tachyarrhythmias can result in diminished time for ventricular filling in diastole (EDV) as well as the loss of the atrial contribution to ventricular diastolic filling (EDV). This ultimately results in lower SV and, thus, CO. These sustained tachyarrhythmias (those that do not result in ventricular fibrillation and SCD) are generally only the cause of cardiogenic shock in the already compromised ventricle. Figure 1 illustrates the effect of a (hemodynamically tolerated) VT on the pressure-volume loop with a reduction of the preload, resulting in a decreased SV and CO (19).

### Main causes of cardiogenic shock related to arrhythmias

Table 1 provides a (non-extensive) overview of the most frequent causes of brady- and tachyarrhythmias associated with CS. Any arrhythmia can be secondary to pre-existing underlying cardiomyopathy (e.g., ischemic or dilated cardiomyopathy) and thus a direct cause of the CS state or the other way around. Indeed, frequent arrhythmias [mainly atrial fibrillation (AF)] can ultimately lead to arrhythmia-induced cardiomyopathy and refractory CS (20). Reports on arrhythmia-induced CS are scarce and probably underrecognized, but Hékimian et al. reported that CS was the first disease manifestation in 60% of their non-ischemic VA ECMO population with recent onset of supraventricular arrhythmia (21).

**Table 1:** Etiologies of bradycardia- and tachycardia-induced cardiogenic shock.

**Bradycardia induced cardiogenic shock (sinus-**,	
**idioventricular-, escape rhythm, …)**	
(Acute) cardiac disease	Ischemia, myocarditis, cardiomyopathies, …
Hypoxia	Pulmonary embolism, acute respiratory distress syndrome, …
Drug induced	Beta blocker intoxication, calcium channel blockers, digoxin, amiodarone, …
Hypothermia	
Device failure	Pacemaker dysfunction, lead fracture, …
Ion disturbances	Hyperkalemia, hypermagnesemia
(Acute) conduction abnormalities	Sinus or atrioventricular disturbances, atrial fibrillation or flutter
Congenital heart or conduction abnormalities	
**Tachycardia induced cardiogenic shock (VF, VT, torsades de pointes, AF**,	
**atrial flutter, …)**	
(Acute) cardiac disease	Ischemia, myocarditis, cardiomyopathies, …
Drug induced	Cocaine, methamphetamine
Ion disturbances	Hypokalemia, hypomagnesemia
Valvular heart disease	
Congenital heart or conduction abnormalities	Brugada syndrome, long-QT syndrome

Acute sustained bradycardia is most frequently related to hypoxia (22), conduction abnormalities, drug intoxications (23), or underlying (ischemic) cardiomyopathy. Sustained tachyarrhythmias can be divided into (more benign) supraventricular tachycardia and (malignant) ventricular tachycardia. AF is the most common sustained supraventricular arrhythmia, and its most important risk factor comes with age (4% of the population over 60 has a sustained episode of AF) (24). In general, supraventricular tachycardias are well-tolerated unless they rise on top of the already compromised ventricle. Arrhythmia-induced cardiomyopathy should be suspected in patients with tachyarrhythmia and dilated cardiomyopathy of no clear etiology (25).

The most frequent malignant tachyarrhythmia is ischemia-induced sustained VT, which can result in ventricular fibrillation (VF), SCD, or CS. VT can be related to a new onset acute myocardial infarction or rising from scar tissue from a previous insult (26). Lethal ventricular arrhythmias have been reported to occur in more than 10% of all acute myocardial infarction cases, and survival in these patients is poor.

### Ventriculo-arterial coupling and uncoupling during an arrhythmic storm

**Figure 2:**
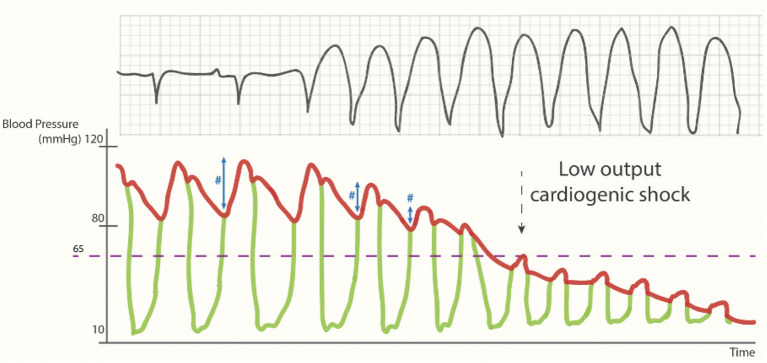
Cardiac output during (unsupported) sustained ventricular tachycardia. Loss of pulse pressure (#) and cardiac output during the arrhythmogenic storm phase [reproduced with permission from (27)].

In a normal cardiac cycle, the LV overcomes the diastolic blood pressure during the isovolumetric contraction phase until the aortic valve opens and the intraventricular blood gets ejected (Figure 2, the left part of the trace; ventriculoarterial coupling). However, during a low output state (e.g., an arrhythmogenic VT storm), the drop in preload and diastolic filling time will eventually result in a drop in pulse pressure and perfusion pressure, ultimately leading to a cardiac arrest phase (Figure 2, the right side of the trace) (27).

**Figure 3:**
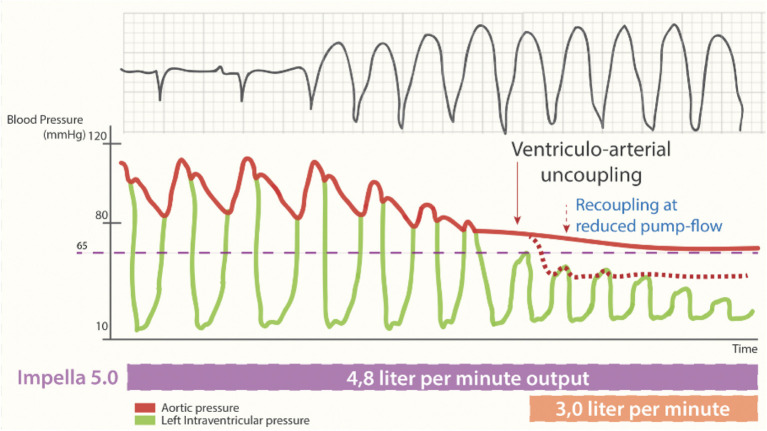
Cardiac output during percutaneous MCS supported sustained ventricular tachycardia. Loss of pulse pressure and cardiac output during the arrhythmogenic storm phase. Ventriculo-arterial uncoupling with sustained output, generated by non-pulsatile output (4.8 L/min) by the Impella-5.0 support and resulting in a systemic blood pressure of 65 mmHg [reproduced with permission from (27)].

This drop in the native cardiac output during an arrhythmic storm can be partly covered by the insertion of a percutaneous mechanical circulatory support device (e.g., an Impella-device), which offers a continuous forward flow of blood from the LV into the aorta. This flow is both afterload sensitive, with end-organ perfusion increasing with lower systemic vascular resistance, and preload dependent, requiring sufficient volume from the right ventricle to operate effectively. If the failing LV can no longer overcome afterload in the new equilibrium of increased mean arterial pressure and reduced preload created by the continuous flow of the percutaneous MCS device, the arterial trace will flatten. This process is called ventriculoarterial uncoupling (Figure 3) (28).

### MCS during arrhythmia-induced cardiogenic shock

Bradycardia-induced CS can often be reversed by positive chronotropic agents or urgent temporary (ventricular) pacing, which leaves physicians a time window for diagnosing and resolving the underlying cause.

Management of patients with CS shock and (supra) ventricular arrhythmias is particularly difficult. Inotropic treatment, such as dobutamine, is recommended for CS but may exacerbate supraventricular tachycardia or accelerate the heart rate in these patients. Antiarrhythmic drugs (e.g., amiodarone) often have negative inotropic effects and may exacerbate the CS state. Electrical reconversions often risk worsening circulatory failure or cardiac arrest in these critically ill CS patients. Therefore, urgent implementation of MCS devices can be an effective way of stabilizing CS patients and allowing physicians a time window of stability to investigate the underlying cause of the arrhythmia and allow therapeutic interventions (e.g., revascularization and semi-urgent ablation) (21). The field of MCS in arrhythmia-related CS is highly unexplored and needs further investigation.

### Left ventricular assist devices and ventricular hemodynamics

Left ventricular assist devices (LVADs) are used to support a failing heart as a temporary means (bridge to recovery and bridge to transplant) or as destination therapy. The interaction between reduced native cardiac function and continuous flow generated by LVAD creates a complex interplay, which may impact the restoration of LV performance.

An elegant *in vitro* study by Viola et al. confirmed how LVAD flow affects intraventricular hemodynamics and pressure. Indeed, the unloading of LV generated by LVAD causes a reduction in ventricular peak systolic pressure, which is related to LVAD output (29). Considering that an increment in intraventricular pressure leads to adverse remodeling of LV myocardium, LVAD, with its unloading effect, may help recover native cardiac function. Recovery of physiological hemodynamic conditions in patients treated with long-term LVAD is still a complex matter of debate with an emerging working hypothesis (30).

## Percutaneous MCS during catheter ablation for ventricular arrhythmias

### Rescue MCS and preemptive MCS during CA for ventricular arrhythmias

Hemodynamic instability, CS, and cardiac arrest are all potential severe complications of CA ablation during electrophysiological studies with a consequent detrimental impact on outcomes and an increased 30-day, 6-month, and 1-year mortality rates (12). A case series of intraprocedural implantation of VA ECMO as rescue therapy for acute hemodynamic deterioration during CA for an electrical storm showed a poor prognosis with high short-term mortality of almost 90%, despite a high rate of CA success (83%) (31).

The dismal prognosis of patients experiencing cardiovascular instability during CA, even after the emergent implantation of MCS, has redirected focus from the stabilization of periprocedural acute hemodynamic decompensation to the identification of preemptive strategies to support high-risk patients. Promising results derived from a single-center study showed lower 30-day mortality (4 vs. 58%) in patients who received pre-emptive insertion of percutaneous LVAD (Impella) compared to patients undergoing rescue insertion of MCS (15).

Although the evidence is still scant, the clinical rationale for preemptive implantation of temporary MCS during high-risk CA of VT is strong. The adequate timing of temporary MCS implantation for high-risk patients (i.e., with structural heart disease, comorbidities, and multiple ICD shocks) undergoing CA of VT is still under debate. However, it is evident that this aspect is of pivotal importance given the high mortality rate of patients requiring emergent implantation of MCS during CA procedures. For this reason, it is crucial to identify, before the procedure, those patients who may experience acute hemodynamic deterioration during the CA of VT.

### PAINESD score and the risk of hemodynamic decompensation

In 2015, Santangeli et al. studied the prevalence and predictors of periprocedural acute hemodynamic decompensation in patients undergoing radiofrequency CA for scar-related VT. In this study, almost one out of 10 patients experienced hemodynamic instability, impacting mortality. The authors identified eight clinical factors predicting periprocedural acute hemodynamic decompensation (PAINESD risk score): advanced age, chronic obstructive pulmonary disease, use of general anesthesia, ischemic cardiomyopathy, heart failure (NYHA classes III and IV), lower LV ejection fraction, presentation with VT storm, and diabetes mellitus (12).

The same study group demonstrated that the percutaneous LVAD implantation prophylactic strategy effectively decreased the incidence of periprocedural acute hemodynamic decompensation. This finding was in synchrony with a reduction in mortality and/or requirement for heart transplantation comparing patients with prophylactic percutaneous LVAD and patients requiring rescue MCS. However, when further analyzing the different categories in the PAINESD score, a considerable benefit in mortality was found in those patients considered at high risk [PAINESD score ≥ 15: hazard ratio (HR) 0.43; 95% confidence interval (CI) 0.21–0.87; *p*-value = 0.02]. Conversely, patients with a predicted low risk of acute hemodynamic decompensation did not experience any benefit (PAINESD score ≤ 8: HR 0.63; 95% CI 0.24–1.66; *p*-value = 0.35) (16).

In their systematic review, Mariani et al. calculated the PAINESD score in the studies published after 2016, showing higher values of the PAINESD score in patients requiring temporary rescue MCS during CA. They confirmed how preemptive implantation of temporary MCS positively influenced the survival rate compared to rescue MCS strategies, particularly in the case of electrical storm. The authors stressed how the PAINESD risk score could be considered a “*robust tool to identify high-risk patients who might benefit from temporary prophylactic MCS during electrophysiological studies*.” They suggested a PAINESD cut-off score of 15 for considering preemptive implantation of temporary MCS, particularly if prolonged VT mapping is required or long phases of unstable VT are expected (17).

### Influence of MCS on the success rate of ablation

Temporary MCS may not only provide cardiovascular support in the case of acute hemodynamic decompensation during CA but may also represent a tool for facilitating the treatment of VT. Some studies have addressed the question of whether temporary MCS influences the success of VT ablation.

Effective ablation of unstable VT with activation and entrainment mapping may be hampered by poor hemodynamic tolerance of induced arrhythmia. In these scenarios, pace and substrate mappings are classically used to find an ablation target while the patient is in sinus rhythm. However, successful ablation is obtained at the expense of scar mapping and ablation (32, 33). In addition, an alternative strategy may be required for those who experience persistent VT after unsuccessful substrate-guided CA, VT related to non-ischemic dilated cardiomyopathies, and VT originating from extensive scars (34).

The feasibility and the advantages of this approach in patients with scar-related ventricular arrhythmias started being assessed in small groups of patients almost 10 years ago. One of the first studies was published by Miller et al. in 2011. They showed how temporary MCS with Impella 2.5 guaranteed end-organ perfusion during prolonged periods of unstable VT, leading to procedural advantages compared to support with an intra-aortic balloon pump (IABP) or no mechanical support (35).

One year later, Bunch et al. analyzed a cohort of high-risk patients with hemodynamically unstable VT undergoing CA guided by activation and entrainment mapping and assisted by Tandem Heart. Despite longer procedure times, the outcomes of acute complications (including death and stroke) and freedom from ICD or therapies for sustained VT were comparable to a matched cohort undergoing substrate mapping without temporary MCS (36).

In a retrospective study, Aryana et al. demonstrated a shorter total radiofrequency ablation time for patients receiving percutaneous LVAD for CA of unstable VT compared to procedures without MCS. That was accompanied by a reduced length of hospital stay (37). The substrate mapping technique is less effective for CA in individuals with ventricular arrhythmias related to non-ischemic cardiomyopathies; in this subset of patients, a combined approach with temporary MCS and activation/entrainment mapping may be particularly beneficial (38).

Despite the differences found in diverse case series, the long-term follow-up did not reveal any statistically significant difference in rates of VT recurrence between patients undergoing CA with and without temporary MCS (39, 40). These findings may be related to heterogenous patient selection and etiologies of arrhythmias in currently available studies (17). Only one study showed how percutaneous LVAD support was associated with a lower composite endpoint of 30-day rehospitalization, redo-VT ablation, recurrent ICD, and 3-month mortality (37).

In the context of hemodynamically tolerated ventricular arrhythmias, the substrate-based CA strategy has proven to have similar acute procedural efficacy and VT recurrence compared with activation and entrainment mapping, with a comparable rate of complications and mortality (41). For those patients who underwent substrate-based CA of unstable VT with arrhythmia recurrence, despite the successful modification of the substrate, ablation guided by activation or entrainment mapping and supported by temporary MCS may represent a reasonable treatment strategy (34).

### Comparison of temporary MCS devices

Different devices, from IABP to VA ECMO, have been proposed as hemodynamic support during CA procedures, thus further increasing the variability between the studies. Each MCS device can guarantee a different level of cardiovascular assistance. Unfortunately, only a few studies have addressed the specific issue of direct comparison between MCS devices.

IABP was proven less effective in providing hemodynamic support than percutaneous LVAD (Impella 2.5) during CA for VT. In a multicentre study, Reddy et al. found that implantation of Impella or Tandem Heart facilitated activation and entrainment mapping of several unstable VTs with fewer rescue shocks compared to patients supported with IABP (40). A retrospective analysis comparing percutaneous LVAD (Impella, Tandem Heart, and VA ECMO) with IABP proved better short-term outcomes in terms of mortality, length of hospital stay, the incidence of acute kidney injury, and 30-day rehospitalization in patients supported by percutaneous LVAD (37). Despite better performance in periprocedural support during CA for unstable VT, no significant differences were found when analyzing intermediate and long-term outcomes, such as VT recurrence and mortality (37).

Impella and Tandem Heart have specific contraindications to their implantation: LV thrombosis, mechanical aortic valve replacement, and ventricular septal defect. These contraindications must be considered when choosing a temporary MCS device. Furthermore, these two devices are burdened with technical limitations related to the requirement of transeptal puncture for Tandem Heart and electromagnetic interference during mapping for Impella (34).

VA ECMO can provide both biventricular and respiratory support, thus allowing its use in extreme conditions of hemodynamic instability, such as cardiac arrest, and as a bridge to recovery or heart transplantation (42, 43). Complete cardiovascular and respiratory support benefits are counterbalanced, especially in the case of femoral percutaneous cannulation, with a retrograde infusion of blood. The consequent LV afterload increase can trigger a vicious cycle leading to increased wall stress and myocardial oxygen consumption (42).

Given its unparalleled capacity to provide end-organ perfusion, VA ECMO represents a useful solution for hemodynamic support in adult patients presenting with electrical storm, VT refractory to antiarrhythmic therapy, and recurrent VF. The combination of hypotension due to refractory VT/VF, cardiac stunning related to repeated shocks, and the frequent requirement for sedation/anesthesia during CA for VT/VF arrhythmic storms may precipitate acute hemodynamic decompensation (34). In the presence of CS related to electrical storm refractory to antiarrhythmic therapy, emergent implantation of VA ECMO may represent a rescue strategy capable of achieving a survival rate after the implantation of 50% (44). However, a smaller case series enrolling 21 patients showed a higher mortality rate (88%) in patients receiving VA ECMO for acute hemodynamic decompensation during CA of VT, despite a high procedural success rate (31).

VA ECMO is the most commonly used temporary MCS device as a rescue strategy for cardiovascular support in children requiring radiofrequency ablation for tachycardia-induced cardiomyopathy (45, 46), for management of hemodynamically unstable primary arrhythmias in newborns and infants (47), and hemodynamic support during acute fulminant myocarditis complicated by arrhythmias (48).

## Impact of permanent ventricular assist devices on arrhythmias

Permanent ventricular assist devices (VADs) represent a therapeutic option for end-stage heart failure as a bridge to heart transplantation or even as “destination therapy” (49). The reported incidence of ventricular arrhythmias after the implantation of LVAD ranges from 20 to 60% (50). If the short-term effects may be negligible, the impact of long-term and recurrent ventricular arrhythmias in patients with long-term LVAD must not be overlooked. In fact, the persistence of ventricular arrhythmia in these patients may cause right heart dysfunction, ultimately leading to hemodynamic compromise (51). From a mortality point of view, the presence of ventricular arrhythmias after LVAD implantation did not affect short-term mortality but significantly increased long-term one (52). Proposed mechanisms for developing ventricular arrhythmias in these patients are multiple and encompass (51, 53): preload alteration with chamber collapse and “suction-related” VT, changes in myocardial electrolyte balance, primary cardiomyopathy, device-related mechanical stimulation, and hypersympathetic state. Predictors of post-operative ventricular arrhythmias after the implantation of LVAD are the history of pre-LVAD ventricular arrhythmias and duration of heart failure (54–56), and the role of underlying cardiomyopathy type is still debated. The findings regarding the onset of ventricular arrhythmias after the implantation of LVAD have been recently summarized in a review (52).

Lin et al. have addressed the role of ventricular arrhythmias in patients with biventricular assist devices (BIVAD) in a retrospective cohort study (57). The prevalence of ventricular arrhythmias in patients treated with BIVAD was high and similar to a propensity-matched LVAD population (46 and 38%, respectively). They also found that patients with sustained ventricular arrhythmias after BIVAD implantation had worse composite outcomes.

## Venoarterial ECMO in arrhythmic storms

Patients with CS refractory to inotropic agents and vasopressors have a poor prognosis, and the VA ECMO offers the ability to restore hemodynamics and prevent end-organ damage. Time to decision and time to initiate VA ECMO is crucial. A narrow “window of opportunity” for rescue VA ECMO intervention exists, beyond which a patient may develop hypoperfusion brain damage, multiorgan failure, reperfusion sepsis, and is too ill to benefit from a temporary MCS (58).

The applications of the VA ECMO in life-threatening arrhythmias encompass arrhythmogenic storm triggered by cardiac ischemia, fulminant myocarditis with ventricular arrhythmias, periprocedural in the cath-lab, accidental hypothermia, and some poisonings (particularly with a concoction of the yew tree needles or the recreational drugs like cocaine or amphetamine) (59). Right ventricular failure may also trigger intractable arrhythmias, like in Ebstein anomaly or other congenital heart diseases, potentiated by positive end-expiratory pressure-induced acute *cor pulmonale*.

In a patient younger than 70 years with cardiac arrest, an ideal therapeutic window for a VA ECMO start (i.e., the time from a collapse to running extracorporeal life support) is within 40 min from the witnessed collapse. The maximum associated delay with acceptable rates of cerebral performance score (CPS) 1–2 is up to 60 min. Crucial is decision-making at 10–15 min of refractory cardiac arrest. That also relates to the expected transfer to the facility or the ECMO to the scene and percutaneous cannulation times of ~14–20 min (60).

The time from collapse to the provision of advanced life support should not be more than 5 minutes and the initial rhythm on the scene associated with a favorable outcome is a shockable VF/VT (60, 61). Other parameters linked to favorable outcomes are time to defibrillation and time to percutaneous coronary intervention in a coronary ischemic event. The contraindications to extracorporeal life support (ECLS) are age above 70 years, unwitnessed cardiac arrest, prolonged time of cardiopulmonary resuscitation (CPR) assuming prolonged time to run ECMO, pre-existing irreversible neurologic, oncologic, or other systemic disease limiting the potential for recovery, a contraindication to systemic anticoagulation, aortic dissection, cardiac tamponade, and severe aortic insufficiency (61).

ECMO has demonstrated its impact on outcomes of cardiac arrest and CS (62). The survival benefits of ECMO in refractory cardiac arrest were demonstrated in the *CHEER (mechanical CPR, hypothermia, ECMO, and early reperfusion) trial*. The investigators reported rates of survival to hospital discharge up to 60% among recipients of ECMO after in-hospital cardiac arrest (IHCA), especially when related to cardiac etiology (63). Acute myocardial infarction or ischemia is the most common cause, accounting for nearly 35–50% of cardiac arrests. Pulseless VT/VF is the initial rhythm in 13–39% of IHCA patients, where temporary MCS and ECMO are used increasingly. The 3-fold increase in the utilization of ECMO in the IHCA over the last decade significantly increased the overall hospital survival from 35.4 to 43.5% (*p* < 0.0001) (64). ECMO yielded more favorable results in patients who suffered IHCA than in out-of-hospital cardiac arrest (OHCA) (60). Recent advances in the care of the OHCA (60) have shown a favorable neurological outcome at 180 days in both study (31.5%) and control (22%) groups related to an established bundle of early intra-arrest transport, invasive assessment, and treatment in both the ECMO and conservative arms of the trial.

The use of VA ECMO during periprocedural arrhythmia is increasing. A substantial mortality benefit was observed among high-risk patients identified with a PAINESD risk score or suffering from electrical storm and treated with preemptive temporary MCS (17). The patients supported predominantly by urgent VA ECMO for periprocedural life-threatening arrhythmias were characterized by older age, more ischemic cardiomyopathies, worse LV ejection fraction, and more comorbidities than the control group. Regardless of unfavorable profiles and the rates of pump failures in the VA ECMO cohort, the rescue ablation successfully prevented recurrences of ventricular arrhythmias and resulted in a comparable 1-year outcome between arrhythmic storms with and without VA ECMO support (65).

Severe accidental hypothermia is associated with ventricular arrhythmias (66–69), and the patient should be referred to the nearest hospital with an ECLS availability. VA ECMO implementation is recommended in severe hypothermia patients (i.e., body temperature <28°C, Swiss hypothermia scale III-IV) and hemodynamic instability defined as ventricular arrhythmias or cardiac arrest (66, 68–70). The call to activate the ECLS pathway or to take a hypothermia patient with a maintained airway and palpable bradyarrhythmia (sinus, junctional rhythm, or atrial fibrillation with slow ventricular response) to the nearest hospital might be challenging. The presence of hypothermia under 30°C significantly limits the chance for cardioversion, either electric or pharmacologically potentiated (66, 69).

A study demonstrated a promising hospital survival of 89% with an outstanding CPS in a case series of patients with severe hypothermia retrieved in a European urban area and treated in an established ECLS and extracorporeal-CPR center. The rewarming requires a short duration of the VA ECMO (median 48 h) with decreasing blood flow and lower sweep gas flow. These are related to an early afterload effect on the heart recovering from hypothermia and hypothermia-related low CO_2_ production (71).

The cannulation should not interfere with the CA techniques if inserted as periprocedural support with a femoro-femoral approach, representing the desirable configuration for periprocedural support or during ECLS.

In all the settings, the return arterial cannula may frequently block the distal leg perfusion, which is, in most centers, solved by routine cannulation of the prograde 6–7F vascular sheath into the superficial femoral artery. The insertion should be as close to the ECMO return cannula as possible because the no-flow segment between them soon becomes a site of thrombus formation.

A minimum cardiac output of 1–2 l/min should be maintained even in circulatory failure supported by the VA ECMO, which would secure LV unloading and prevent intracardiac thrombus formation. If this is not possible, the available methods of unloading are Impella, a surgical vent of the LV either through the mitral valve and left atrial auricle or transseptal approach, or to a certain degree, also with the IABP (72, 73).

Details regarding VA ECMO configuration, hemodynamics, and complications are beyond the scope of the current review and are detailed elsewhere.

## Stellate ganglion blockade during MCS

As detailed above, MCS is an effective strategy to rest the heart and support the circulation when it is compromised by ineffective cardiac contraction during an arrhythmic storm. Although sinus rhythm may be restored after the initiation of VA ECMO in the ECLS setting, intractable ventricular arrhythmias may persist despite full circulatory support. In a recent publication of patients treated with MCS during arrhythmic storm, no differences between survivors and non-survivors were noted according to the use of antiarrhythmic drugs (amiodarone, lidocaine, and/or electrolyte adjustment) (74).

The percutaneous blockade of the stellate ganglion has been described in different populations, including in patients with electrical storm on MCS. It is a minimally invasive (either blind or ultrasound-guided) technique that has been demonstrated to relieve ventricular arrhythmic burden in a remarkable rate of patients when all other pharmacological therapies failed without serious complications (75–77).

## Arrhythmias in COVID-19

Large studies have reported an overall prevalence of arrhythmias after SARS-CoV-2 infection that ranges from 10 to 20%, although the incidence is greatly increased in individuals with severe disease. Arrhythmias are supraventricular in most of the critically ill patients with COVID-19. However, 40% of the overall arrhythmias are ventricular tachyarrhythmias, bradyarrhythmias, and conduction defects, which are associated with remarkably high mortality (78).

Inflammatory cytokines, particularly TNF, IL-1, and IL-6, may exert significant arrhythmogenic effects *via* several mechanisms, including complex modulatory activities on the expression and function of specific ion channels and gap junction-forming connexins, cytochrome system inhibition, and structural remodeling by activating the myofibroblast-driven synthesis of extracellular matrix responsible for cardiac fibrosis (79). Additionally, oxygen mismatch and sympathetic activation may also work as triggers for arrhythmias. In a large cohort of patients hospitalized with COVID-19, an independent association between infection status and QTc prolongation and a direct correlation between IL-6 levels and QTc interval were shown (80).

During the COVID-19 pandemic, a provision of ECMO services has been offered, underscoring the need to select cases that may benefit from ECMO placement. The use of MCS for CS was rather low during the pandemic, although the number of cardiac arrests was higher, at least during the “first wave” (81). Cardiac abnormalities also prolong the long-COVID syndrome with arrhythmias, which are part of the symptoms of post-acute sequelae (82). The burden of heart failure with all the relevant clinical consequences, including cardiac dysautonomia, is expected in patients who experienced severe COVID-19 infection and have recovered.

## Final considerations

MCS is an effective strategy to support hemodynamics in patients with an arrhythmic storm. The choice of the device should be driven by the team experience, clinical setting, and amount of cardiocirculatory support required. Collateral therapies, such as titrated drugs, metabolic adjustment, and percutaneous stellate ganglion blockade, are essential to stabilize the arrhythmic burden and need further investigation.

## Author contributions

All authors listed have made a substantial, direct, and intellectual contribution to the work and approved it for publication.

## Funding

This manuscript adheres to the Ricerca Corrente Fondazione Policlinico San Matteo IRCCS (RCR-2021-23671212), from which the publication fees have been provided.

## Conflict of interest

The authors declare that the research was conducted in the absence of any commercial or financial relationships that could be construed as a potential conflict of interest.

## Publisher's note

All claims expressed in this article are solely those of the authors and do not necessarily represent those of their affiliated organizations, or those of the publisher, the editors and the reviewers. Any product that may be evaluated in this article, or claim that may be made by its manufacturer, is not guaranteed or endorsed by the publisher.
